# Adverse reactions induced by MDT/WHO (Rifampicin+Clofazimine+Dapsone) and ROM (Rifampicin+Ofloxacin+Minocycline) regimens used in the treatment of leprosy: a cohort study in a National Reference Center in Brazil

**DOI:** 10.3389/fphar.2024.1346169

**Published:** 2024-03-07

**Authors:** Isadora Costa Celestino, Douglas Eulalio Antunes, Diogo Fernandes Santos, Victor Lemos Gimenes, Fabiane Mian de Souza, Isabela Maria Bernardes Goulart

**Affiliations:** ^1^ Post-Graduation Program in Health Science, Faculty of Medicine, Federal University of Uberlândia, Uberlândia, Brazil; ^2^ National Reference Center for Sanitary Dermatology and Leprosy, Clinics’ Hospital, Faculty of Medicine, Federal University of Uberlândia, Uberlândia, Brazil; ^3^ Faculty of Medicine, Higher School of Health Sciences, Federal District Health Department, Brasília, Brazil; ^4^ Faculty of Medicine, Federal University of Uberlândia, Uberlândia, Brazil

**Keywords:** leprosy, drug-related side effects and adverse reactions, Rifampicin, Dapsone, Clofazimine, Ofloxacin, Minocycline, cohort study

## Abstract

**Background:** Recommended standard treatment for leprosy is multidrugtherapy (MDT/WHO), consisting Rifampicin+Dapsone+Clofazimine. Other medications are recommended in cases of resistance, adverse reactions and intolerances, including ROM regimen, Rifampicin+Ofloxacin+Minocycline. Therefore, pharmacovigilance is an important tool in understanding these adverse drug reactions (ADRs), supporting pharmacotherapy management and medication safety. This study seeks to evaluate ADRs comparing two therapeutic regimens, MDT and ROM, used in treatment of patients with leprosy, analyzing prognostic factors regarding risk and safety.

**Methods:**A retrospective cohort study was performed by assessing medical records of 433 patients diagnosed with leprosy from 2010 to 2021 at a National Reference Center in Brazil. They were subject to 24 months or more of treatment with MDT or ROM regimens. ADR assessments were analyzed by two experienced researchers, who included clinical and laboratory variables, correlating them with temporality, severity and the causality criteria of Naranjo and WHO.

**Results:** The findings observed an average of 1.3 reactions/patient. Out of individuals experiencing reactions, 67.0% (69/103) were utilizing MDT/MB, while 33.0% (34/103) were using ROM. The median time for ADR of 79 days for MDT and 179 days for ROM. In first reaction, Dapsone was the most frequently involved medication; the most affected system was hematopoietic. As compared to Clofazimine, results indicated that use of Dapsone was associated with 7% increased risk of ADR occurrence (HR: 1.07; *p* = 0.866). Additionally, Rifampicin was linked to 31% increased risk of ADRs (HR: 1.31; *p* = 0.602); and Ofloxacin showed 35% elevated risk (HR: 1.35; *p* = 0.653). Conversely, results for Minocycline indicated 44% reduction in the risk of ADRs (HR: 0.56; *p* = 0.527), although statistical significance was not reached. The use of MDT conferred 2.51 times higher risk of developing ADRs in comparison to ROM.

**Conclusion:** The comparison between MDT and ROM revealed that MDT caused more ADRs, and these reactions were more severe, indicating less safety for patients. Dapsone was the most common medication causing ADRs, followed by Rifampicin. The combination with Clofazimine was associated with an additional risk of ADRs, warranting further studies to confirm this hypothesis. Given the high magnitude of ADRs, healthcare teams need to monitor patients undergoing leprosy treatment with focus on pharmacovigilance.

## 1 Introduction

Leprosy is caused by the etiological agents *Mycobacterium leprae* and *Mycobacterium lepromatosis* (Belachew et al., 2019), and one of the transmission routes being direct contact with another infected human (Ploemacher et al., 2020). Diagnosis is challenging due to the long incubation period of the *bacillus*, which can take up to 20 years after contact for the appearance of the first signs and symptoms (Pfaltzgraf et al., 1985; Goulart et al., 2008).

The disease affects nerves due to the *bacillus*’ tropism for Schwann cells (SC) in the peripheral nervous system (PNS), initially causing increased, decreased, or absence of tactile sensation (Ng et al., 2000; Mungroo et al., 2020). It causes irreversible physical and motor damage, with these being the main contributors to the stigma of the disease. Other signs and symptoms depend on the clinical form and include hypopigmentation, pruritic lesions, hair loss, macules, neuropathies, ocular impairments, including lagophthalmos, nasal obstructions, epistaxis, septal perfusion, and others (Maymone et al., 2020; Chen et al., 2021).

The operational classification for treatment purposes considers paucibacillary (PB), when there are up to five skin lesions with negative intradermal bacilloscopy, and multibacillary (MB) with the presence of six or more skin lesions or positive intradermal bacilloscopy (Brasil, 2017). The clinical classification (Ridley and Jopling, 1966) allows the characterization of the disease spectrum, ranging from a vigorous cellular immune response in the Tuberculoid form (TT), through the unstable Borderline group, including Borderline-Tuberculoid (BT), Borderline-Borderline (BB), Borderline-Lepromatous (BL), and the high bacillary load and low cellular immunity form, Lepromatous (LL). Additionally, a subclassification of Lepromatous is called subpolar Lepromatous, considered a degraded borderline (Ridley and Job, 1985).

Brazil is the second country with the highest number of leprosy cases according to the WHO, accounting for approximately 79.0% of new diagnoses in 2019, along with India and Indonesia (WHO, 2020). For treatment, WHO recommended the use of multidrug therapy (MDT) in 1981, consisting of Dapsone (DDS), Clofazimine (CFZ), and Rifampicin (RFM). Currently, the regimen can be used for both PB and MB patients. Thus, the regimen used in adults diagnosed with MB leprosy (MDT/MB) is 600 mg of RFM and 300 mg of CFZ monthly, with daily doses of 50 mg of CFZ and 100 mg of DDS, from 12 to 24 doses (WHO, 1998; WHO, 2018).

However, over the years, drug resistance, therapeutic failures, and relapses in leprosy treatment have been observed, along with adverse reactions, including intolerances and hypersensitivities to MDT, making use and patient adherence challenging. Therefore, new effective drugs for treatment have been studied, such as Ofloxacin (Ji et al., 1994), Minocycline (Gelber et al., 1994), Clarithromycin (Gunawan et al., 2018), and other quinolones, such as Moxifloxacin (Pardillo et al., 2008; Franco-Paredes et al., 2022), and Levofloxacin (Dhople et al., 1995). Thus, a new alternative regimen with good acceptance has been called ROM (WHO, 1998), an association of Rifampicin (RFM) 600 mg, Ofloxacin (OFX) 400 mg, and Minocycline (MNC) 100 mg in monthly doses, recommended for use in intolerances or contraindications to CFZ and DDS, with a minimum duration of 12 months for PB and 24 months for MB (Brasil, 2016).

However, like MDT, the ROM regimen can also cause adverse reactions, which should be assessed within the concept of pharmacovigilance: “the science and activities related to the detection, assessment, understanding, and prevention of adverse reactions or any other possible drug-related problem,” ensuring patient safety and treatment quality (WHO, 2006). Thus, adverse reactions to drug use (ADRs) are: “harmful, unintended reactions that occur at doses normally used in humans” (WHO, 1973). However, for the drug to be related to the suspicion of a reaction, it is necessary to observe causality linked to the drug. To do this, one must consider not only previous studies on the drug but also temporality and, if possible, clinical and laboratory variables (OPAS, 2011). For this purpose, there are criteria, the most well-known being the Naranjo et al. (1981) Algorithm and the WHO Causality (OPAS, 2011). Both allow for an assessment of the plausibility of the medication causing the reaction, considering several variables such as reintroduction of the medication (rechallenge) or even the gradual withdrawal of the dose (dechallenge).

In the new global strategy from 2021 to 2030, towards zero leprosy, WHO emphasized the need for pharmacovigilance in leprosy, so that adverse reactions are monitored and identified, enabling management and improvements in the pharmacotherapy of patients affected by the disease (WHO, 2020). Therefore, Brazil being the second highest in number of leprosy cases globally, a study to elucidate the ADRs of the main drug regimens is necessary to provide information to healthcare teams, enabling appropriate and timely management for treatment adherence and consequently reducing *M. leprae* transmission.

Thus, the aim of this present study was to assess adverse reactions to MDT/MB and ROM regimens presented by patients treated at a Leprosy Reference Center in Brazil over a 12-year period.

## 2 Materials and methods

### 2.1 Study design

The present study is a retrospective cohort that analyzed secondary data from medical records of 449 patients diagnosed with leprosy who underwent treatment with MDT/MB and ROM regimens with 24 doses or more. The study covered the period from January 2010 to December 2021 at CREDESH-HCU-UFU-EBSERH (National Reference Center in Sanitary Dermatology and Leprosy of the Clinical Hospital of the Federal University of Uberlândia). Patients included in the study had documented adverse drug reactions in their medical records or showed significant laboratory findings. Upon temporal correlation, there was plausibility that the medication was the cause of the adverse reaction.

### 2.2 Sampling methods

The method employed to enroll patients in this retrospective cohort study was quota sampling, based on a therapeutic schema that helped the establishment of two subgroups according to the prescribed medication: MDT or ROM.

### 2.3 Ethics statement

This study involving human participants were reviewed and approved by the local research ethics committee CAAE: 46768321.5.0000.5152 (UFU), with informed consent being waived, as it is a study that involves retrospective data from patients who have interrupted or completed treatment.

### 2.4 Inclusion and exclusion criteria

The inclusion criteria encompassed patients diagnosed with leprosy who underwent treatment with MDT/MB or ROM, receiving a minimum of 24 doses between January 2010 and December 2021 at CREDESH-HC-UFU- EBSERH. Exclusion criteria applied to patients under 15 years of age, pregnant individuals, or those who experienced adverse reactions to other medications concurrent with the use of MDT/MB and ROM.

As a result, medical records of 449 patients were analyzed, and 16 were excluded: 14 due to being under 15 years of age and two due to pregnancy.

### 2.5 Data collect

For diagnosis, patients were examined by leprosy-specialized physician, who used clinical and laboratory data in treatment monitoring. Consequently, participants were already under monthly observation at CREDESH-HC-UFU-EBSERH by a multidisciplinary team comprising of physician, nurses, physical therapist, pharmacists and other healthcare professionals. Once leprosy was confirmed, the physicians prescribed the treatment, and the patient received the prescribed medications from the Reference Center’s pharmacy.

Throughout the treatment, patients underwent laboratory tests at the Clinics’ Hospital of Federal University of Uberlândia. These tests were conducted at the beginning and end of the treatment and every 3 months (Supplementary Table S1). If a patient showed any possibility of ADRs the medical team requested additional laboratory tests to monitor the reaction and the outcome after clinical management. This could involve suspension and gradual withdrawal of the dose (dechallenge) and latter reintroduction of the medication (rechallenge), or management with pharmacological or non-pharmacological measures.

For the study, clinical and laboratory data were collected from the included participants, both in physical and electronic medical records. Suspected adverse reactions documented in the records were assessed by two experienced researchers, who correlated them with the clinical and laboratory findings. Patient records were also analyzed for the treatment start date, outcome date or intermediate period (date of ADR occurrence), medication suspension date or management, and when the participant showed clinical and laboratory improvement of the reaction.

### 2.6 Assessment of adverse drug reactions

While collecting all the information described above, data such as epidemiological variables, like as age, sex, and ethnicity were analyzed. Clinical variables observed included operational classification (PB/MB), multibacillary clinical forms (BT, BB, BL, and LL), patient history (previous conditions and habits, use of other medications, and prior adverse reactions), initial and final treatment regimens and presented adverse reactions.

The data, encompassing reactions affecting various systems such as hematopoietic, hepatic, renal, dermatological, nervous, gastrointestinal, ophthalmic, musculoskeletal, and systemic, were meticulously analyzed. The number of Adverse Drug Reactions (ADRs) during treatment, clinical and/or laboratory findings, severity, and time of presentation until resolution were assessed by experts in pharmacology. The analysis followed physician diagnostics described in medical records and employed established instruments, including the WHO Causality and the Naranjo Algorithm (1981).

### 2.7 Statistical analysis

The normality of continuous variables was assessed using the D’Agostino-Pearson test. The binomial test was employed to analyze the association between MDT/MB and ROM groups and factors related to demographic and clinical characteristics of patients affected by an adverse drug reaction. The Friedman test was conducted to compare differences between medians of three paired results concerning hemoglobin concentrations at different treatment periods.

Furthermore, prognostic factors associated with adverse drug reactions were analyzed through Kaplan-Meier survival curves. Multivariate analysis of prognostic factors associated with ADRs was carried out using time-dependent Cox regression with proportional hazards (hazard ratio).

Statistical analysis was performed using the Statistical Package for the Social Sciences (SPSS) version 22.0 software (IBM, Armonk, NY, United States), considering a significance level of 5%.

## 3 Results

### 3.1 Epidemiological data

Records of 433 participants were analyzed: 43% (186/433) initiated treatment with MDT medication regimen, while 57% (247/433) started with ROM regimen. For age criteria, they were divided into age groups: 15 to 19 (19/433), 20 to 29 (26/433), 30 to 39 (63/433), 40 to 49 (89/433), 50 to 59 (110/433), 60 to 69 (70/433), 70 to 79 (40/433), and 80 or older (16/433). Regarding sex, 39.5% were women (171/433), and 60.5% were men (262/433).

Patients in MDT regimen, 6.5% (12/186) were Subpolar Lepromatous, 8.6% (16/186) Borderline-Borderline, 10.2% (19/186) Borderline-Lepromatous, 24.7% (46/186) Borderline-Tuberculoid, and 50% (93/186) were Lepromatous. Additionally, 33.3% (62/186) were women, and 66.7% (124/186) men, with 31.7% (59/186) being 60 years or older.

For patients on the ROM regimen, 2.0% (5/247) were Subpolar Lepromatous, 7.2% (18/247) Borderline-Lepromatous, 16.1% (40/247) Borderline-Borderline, 38.1% (94/247) Borderline-Tuberculoid, and 36.4% (90/247) Lepromatous. Moreover, 44.1% (109/247) were women, and 55.8% (138/247) were men, with 28.3% (70/247) being 60 years or older.

### 3.2 Participants who presented ADRs

The incidence of ADRs were observed in 23.8% (103/433) of the patients. Among these, 42.7% (44/103) were women, and 57.3% (59/103) men. Regarding the clinical form, 39.8% (41/103) were Lepromatous, 14.5% (15/103) Subpolar Lepromatous, 11.6% (12/103) Borderline-Lepromatous, 5.8% (6/103) Borderline-Borderline, and 28.2% (29/103) Borderline-Tuberculoid. Of the participants, 72.8% (75/103) were under 60 years old and 27.2% (28/103) were 60 or older. The skin color with the highest number of participants was white, accounting for 45.6% (47/103), followed by brown, which constituted 39.8% (41/103) of the total.

In addition to leprosy, 24.2% (25/103) of the patients had a total of 38 comorbidities. Among them, 16% (4/25) had more than two comorbidities, including hypertension (14/38), type 2 diabetes mellitus (8/38), hypothyroidism (4/38), chronic kidney disease (3/38), iron-deficiency anemia (2/38), diabetic nephropathy (1/38), fibromyalgia (1/38), chronic hepatitis (1/38), chronic obstructive pulmonary disease (COPD) (1/38), type 1 diabetes mellitus (1/38), dyslipidemia (1/38), and clinical depression (1/38). Patients using other medications, either for other health conditions or for leprosy reactions, totaled 34.9% (36/103).

Among those affected by reactions, 67.0% (69/103) were using MDT/MB and 33.0% (34/103) were using ROM. Of the participants, 77.6% (80/103) experienced only one ADR, 17.4% (18/103) had two ADRs, and 4.8% (5/103) had three or more. A total of 134 adverse reactions were observed in both regimens, averaging 1.3 reactions per patient. In this article, only the first adverse reactions presented by patients will be discussed.

Concerning the severity of the first adverse reaction, 42.7% (44/103) were considered mild, 26.2% (27/103) moderate, and 31.1% (32/103) severe, with one leading to a fatal outcome. Additionally, 36.0% (37/103) were managed with the use of other medications, and in 63.1% (65/103), the medication suspected to have induced the reaction was discontinued.

DDS caused the most ADRs in the first event, totaling 51.4% (53/103). RFM, used in both regimens, caused 33.0% (34/103) of ADRs, but 70.5% (24/34) were associated with the ROM regimen, while 14.4% (10/69) were associated with the MDT/MB regimen. OFX caused 7.7% (8/103) of the reactions, while MNC caused 1.9% (2/103), both from the ROM regimen. CFZ was responsible for 5.8% of total reactions (6/103).

Regarding the World Health Organization (WHO) Causality Assessment for the first ADRs in the MDT/MB regimen, 55.0% (38/69) were classified as probable, 26.0% (18/69) possible, 10.1% (7/69) doubtful, and 8.6% (6/69) confirmed. In the ROM regimen, 58.8% (20/34) were possible, 17.6% (6/34) confirmed, 11.8% (4/34) probable, 8.8% (3/34) doubtful, and 2.9% (1/34) conditional.

In the Naranjo Causality Assessment, for the first ADRs in the MDT/MB regimen, 56.5% (39/69) were classified as probable, 30.4% (21/69) possible, 7.2% (5/69) defined, and 5.7% (4/69) doubtful. In the ROM regimen, 50.0% (17/34) were possible, 26.4% (9/34) probable, 17.6% (6/34) defined, and 5.9% (2/34) doubtful.

During the first adverse reaction, the affected systems were as follows: 36.8% (38/103) hematopoietic, 21.3% (22/103) gastrointestinal, 17.4% (18/103) dermatological, 12.6% (13/103) nervous, 5.8% (6/103) systemic, 2.9% (3/103) renal, 0.9% (1/103) hepatic, 0.9% (1/103) musculoskeletal, and 0.9% (1/103) ophthalmic.

Withing reactions affecting the dermatological system, 22.2% (4/18) were erythroderma, 44.4% (8/18) generalized rash, 11.1% (2/18) pruritic rash, 5.5% (1/18) itchy scalp, 5.5% (1/18) urticaria, and 11.1% (2/18) xeroderma. Cases affected by erythroderma were using MDT/MB, and DDS was the suspected medication, which was discontinued in all cases. For generalized rash, the most suspected causative drugs were RFM (50.0%; 4/8), OFX (37.5%; 3/8), and DDS (12.5%; 1/8). Itchy scalp and urticaria were associated with the use of MNC, confirmed by rechallenge. Only two cases of xeroderma were recorded, associated with the use of CFZ, which was suspended.

Among the gastrointestinal reactions, 13.6% (3/22) were diarrhea, 54.5% (12/22) epigastralgia, 27.2% (6/22) nausea and 4.5% (1/22) constipation. DDS (1/3) and RFM (2/3) were associated with diarrhea. Epigastralgia was related to the use of DDS (1/12) and RFM (11/12). Nausea was associated with the use of DDS (2/6), CFZ (1/6), OFX (1/6), and RFM (2/6). One case of constipation was related to the use of CFZ. Overall, these reactions were managed with pharmacological measures without discontinuing the medications.

Hematopoietic reactions: 86.8% (33/38) were hemolytic anemia, with DDS causing 96.9% (32/33) of these, and Rifampicin causing 3.0% (1/33). Whenever a patient was suspected of hemolytic anemia due to Dapsone, rechallenge tests and dose reductions were performed. These interventions led to the suspension of medications in all cases (32/32) of this research due to a persistent decrease in hemoglobin levels even after Dapsone dose decrement. Regarding anemia caused by Rifampicin, as causality was considered doubtful, the medication was not discontinued, and the reaction was observed over months through routine tests.

In cases of hemolytic anemia caused by DDS (32/33), both sexes showed significant differences in hemoglobin levels between diagnosis and when the patient presented the ADR, according to Dunn’s multiple comparison test (*p* < 0.0001). Men who started the treatment with a hemoglobin of 12.9 g/dL and women with a hemoglobin of 12 g/dL were predisposed to hemolytic anemia onset (*p* < 0.0001). The average time for this specific reaction to occur was 163 days, with an average hemoglobin decrease of 2.8 g/dL. During the ADR, men (25/32) had an average decrease of 2.9 g/dL, ranging from 0.8 to 5.6 g/dL (*p* < 0.0001); while for women (7/32), there was an average decrease of 2.5 g/dL, ranging from 1 to 3.8 g/dL (*p* = 0.0013).

Other hematopoietic reactions included aplastic anemia (3/38) and thrombocytopenia (2/38). In all cases of aplastic anemia (3/3) DDS was the suspected drug, which was discontinued and replaced with OFX, leading to improved levels. In thrombocytopenia, the suspected drug (2/2) was RFM, part of the MDT/MB regimen, which was discontinued and replaced, in one case, with OFX and in the other case, with the MCM regimen (Minocycline+Clarithromycin+Moxifloxacin).

In the hepatic system, the only reaction (1/103) was drug-induced hepatitis due to DDS use, leading to a switch from the MDT/MB regimen to ROM. For the musculoskeletal system, muscle weakness was the only reaction (1/103) related to RFM use. However, upon causal analysis, it was deemed doubtful and the reaction was managed without discontinuing the medication.

Reactions envolving the nervous system, 7.8% (1/13) were anxiety, 23.0% (3/13) headache, 46.2% (6/13) insomnia, and 23.0% (3/12) dizziness. During anxiety reports, the participant was using MDT/MB, and dechallenge/rechallenge tests with DDS were conducted, leading to the probable identification of DDS as the cause of the reaction and resulting in medication suspension. In cases of headaches, RFM was the suspected drug, with 33.3% (1/3) in the MDT/MB regimen and 66.7% (2/3) in the ROM regimen. However, these were managed without discontinuation of the antimicrobial. In insomnia reports, there was a relationship with the use of OFX (4/6), DDS (1/6), and RFM (1/6), all managed with non-pharmacological measures such as sleep hygiene and pharmacological measures such as the use of hypnotic/sedative medications. Cases of dizziness (3/12) were all related to the use of the MDT/MB regimen, both with DDS (2/3) and CFZ (1/3). However, only in one case DDS was suspended after a rechallenge.

Only one eye reaction, xerophthalmia (1/103), was recorded, related to the use of CFZ. Management involved the use of lubricating eye drops, with improvement without discontinuation of the medication. In renal adverse reactions, 66.7% (2/3) were acute renal failure and 33.3% (1/3) interstitial nephritis. All these cases implicated RFM as the suspect, and during the reports of acute renal failure, one was considered doubtful and the other as proven after a rechallenge test. Despite one case of doubtful causality in renal failure, the team preferred to discontinue the medication. The patient diagnosed with interstitial nephritis had the probable cause attributed to the use of the antimicrobial. As with the other two cases affecting the renal system, RFM was discontinued.

In systemic reactions (6/103), 33.3% (2/6) were flu-like syndrome, with RFM being the only associated drug. In one case, the participant used MDT/MB and switched RFM to OFX, and in the other case, the MDT/MB regimen was changed to MCM. Sulfone syndrome (SS) was observed in 50.0% (3/6) of cases, caused exclusively by DDS, with the regimen being discontinued and replaced with ROM (2/3) and MCM (1/3). In 16.7% (1/6), anaphylaxis was associated with RFM during the first supervised dose of the MDT/MB treatment, where upper airway obstruction and angioedema were observed, not responding to team management, leading to death and being the only lethal case in the study.

### 3.3 Survival analysis and Cox’s proportional hazards

The variables: clinical form, sex, skin color, comorbidities, age, and the number of concurrently administered medications with MDT did not represent prognostic factors associated with ADRs (Log Rank; Breslow; Tarone-Ware, *p* > 0.05). On the other hand, the comparison between survival curves for conventional and alternative treatment regimens indicated MDT/MB as a poor prognostic factor for the occurrence of ADRs (Log Rank, *p* = 0.008; Breslow, *p* = 0.002; Tarone-Ware, *p* = 0.002). The median time for the occurrence of ADRs was 79 days for those using MDT/MB versus 179 days for participants under the ROM regimen.

Furthermore, survival analysis stratified by the type of medication confirmed the detection of DDS, CFZ and RFM use as poor prognostic factors for the occurrence of ADRs (Log Rank, *p* = 0.019; Breslow, *p* = 0.055; Tarone-Ware, *p* = 0.031), as their median times for the outcome were 63, 115, and 154 days, respectively, shorter than those found in drugs like OFX (245 days) and MNC (470 days).

The proportional hazards model (hazard ratios) through time-dependent Cox regression, demonstrated, through the multivariate model, that patients under the MDT/MB therapeutic regimen showed a 2.51 times higher risk of ADRs occurrence when compared to those under the ROM regimen (*p* = 0.029).

**FIGURE 1:**
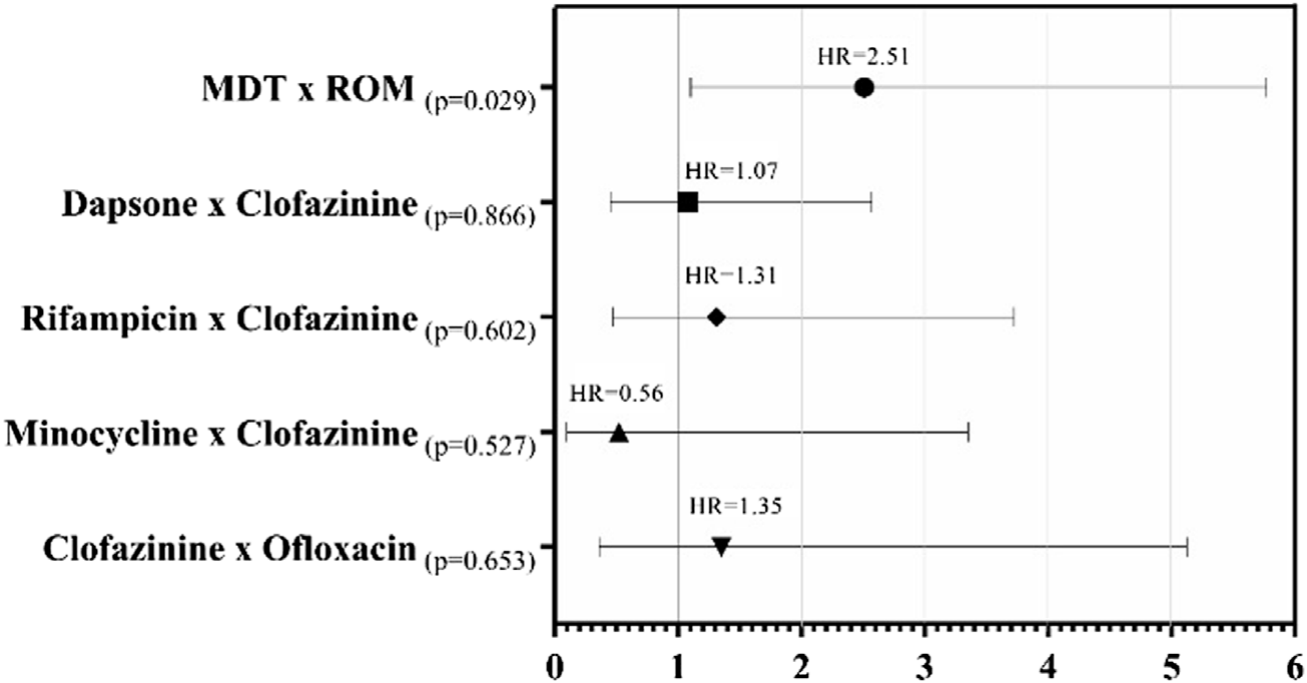
Forest plot for Cox proportional hazards of patients who presented ADRs, with the Kaplan-Meier hazard ratio (HR) of the time to presentation of the first adverse occurrence according to the medication used.

Although other factors, when stratified, did not show prognostic validity in a multivariate model when compared with CFZ, there was an increased risk of 7% with the use of DDS in the regimen for ADR occurrence (HR: 1.07, *p* = 0.866). Furthermore, RFM attributed a 31% increase in the risk for ADRs (HR: 1.31, *p* = 0.602) and 35% with Ofloxacin (HR: 1.35; *p* = 0.653). In contrast, the results for MNC showed that this medication reduced the risk of ADRs in 44% of patients (HR of 0.56; *p* = 0.527) compared to CFZ (Figure 1).

## 4 Discussion

The majority of the study’s participants were men under 60 years old with clinical form LL, which is expected due to the inclusion criteria, requiring a minimum of 24 doses of treatment. This age and sex profile was also observed in the Brazilian census from 2017 to 2021, where 55.7% of cases were male, with a prevalence in the age group of 50–59 years, totaling 23,192 diagnoses out of 119,698 (Brazil, 2023).

Regarding comorbidity profiles, a significant portion had cardiovascular diseases (primary hypertension) and endocrine conditions (type 2 diabetes mellitus and hypothyroidism). It is important to note the other clinical conditions and use of medications as how they can affect ADRs presentation, causing confusion during causality analysis (Magro et al., 2012).

Skin color as a variable for the occurrence of ADRs did not affect the quantity or severity, showing no statistically significant difference (*p* > 0.001). Even though there’s no difference in the present study, articles relate an individual’s skin color to an increased chance of presenting ADRs, mainly those linked to gene alleles. HLA-B*13:01 is an example, leading to a higher predisposition to develop Dapsone Hypersensitivity Syndrome (DHS), known as Sulfone Syndrome (SS), mainly present in Chinese, Japanese, Indian, and Southeast Asian populations (Hoogeven et al., 2016; Liu et al., 2019). The DHS is a type IV-b hypersensitivity reaction characterized as a Drug Reaction with Eosinophilia and Systemic Symptoms (DRESS), mediated by T cells (TH2) that release cytokines and chemokines such as IL-4, IL-5, and IL-13, activating and recruiting eosinophils (Coombs and Gell, 1968; Cabañas et al., 2020). Therefore, patients with known family histories or populations with the potential presence of this gene should avoid the use of Dapsone.

The leprosy treatment regimen that showed the most ADRs was MDT/MB, even though it was not the most commonly used regimen in the study population. This was confirmed by observing the Hazard Ratio (HR), which listed in the analyses that this regimen has a 2.51 times higher chance of leading to the development of an ADR. MDT/MB was more associated with reactions in the hematopoietic system (*p* < 0.0001), the development of severe reactions (*p* < 0.0001), and more than one ADR during treatment (*p* = 0.020). Additionally, this regimen had a greater association with reactions of probable causality, according to the Naranjo Algorithm and the WHO Causality (*p* = 0.004). DDS was the drug that caused the most ADRs, 51.4%, and had the shortest average time to occurrence, 63 days after the start of use.

A previous study conducted at CREDESH-HC-UFU with 187 leprosy patients using MDT/MB observed 37.9% of adverse reactions, with 70.8% (80/113) of reports linked to the use of DDS, showing dermatological, hematopoietic, gastrointestinal, nervous, and musculoskeletal effects, with a predominance of epigastralgia and hemolytic anemia (Goulart et al., 2002).

Guragain et al. study (2017), the average time of appearance of DDS-induced ADRs was 3–21 weeks. Similar to the present study, reactions in the dermatological and hematopoietic systems were observed, such as exfoliative dermatitis (8/37), unspecified skin rash (4/37), hemolytic anemia (5/37), jaundice (14/37), fever, and headaches (4/37), Toxic Epidermal Necrolysis (TEN) (1/37), and agranulocytosis (1/37).

Regarding hemolytic anemia, in the present study, 32% (33/103) presented this ADR, with an average hemoglobin decrease of 2.8 g/dL, while Franco (2014), changes were seen in 16.8% (20/119), with an average decrease of 2.35 g/dL. It was also emphasized that DDS was the main suspect drug, and the use of polychemotherapy alone led to an average decrease in hemoglobin of 1.94 g/dL. This information should be highlighted in the management of patients undergoing MDT/MB.

The avarege time for the other drugs analyzed in the study, CFZ was found to be 115 days (approximately 4 months), but no studies were identified for comparison of this data. Additionally, although not statistically significant, the use of CFZ indicated an increased risk of adverse drug reactions (ADRs) when combined with DDS, while MNC reduced this risk. However, no studies were found that provided a basis for comparing how CFZ elevates this risk and, when combined with MNC, leads to a reduction in the risk of ADRs. Nevertheless, more studies with a larger sample size are needed to confirm this hypothesis.

CFZ caused 5.8% (6/103) of total adverse reactions, including constipation, xerophthalmia, dizziness, nausea, and xeroderma. These ADRs were also observed in Maia et al. (2013), who noted hyperpigmentation in all participants (37/37) and ichthyosis in 21.6% (8/37), both related to CFZ use. Hwang et al. (2014), a systematic review concluded that CFZ is the main cause of skin and gastrointestinal reactions. In a previous study with 119 patients (Franco, 2014), CFZ was the main drug in the MDT causing dermatological reactions, with a frequency of 70.0% for xerosis and 65.5% for skin pigmentation. However, this study also emphasized CFZ as a cause of xerophthalmia, affecting 46.2% of participants. According to the medication’s own label (Novartis, 2016), skin pigmentation can occur in 75%–100% of patients within a few weeks of treatment.

Analyzing the experience of this Reference Center and the collected data, it was concluded that CFZ-induced ADRs are poorly known and investigated by the team, such as gastrointestinal changes caused by the deposition of CFZ crystals in the submucosa and an increased QT interval with arrhythmias presence. This is concerning because, according to Risgaard et al. (2016), the use of at least one QT interval-altering medication in the last 90 days was seen in 58% of patients who experienced sudden death, with antibiotics linked to 16% of those cases. Thus, after analyzing these data, training was conducted with the team, emphasizing the need to record these ADRs.

In a previous study by our group (Goulart et al., 2002) with 187 participants had 113 identified reactions and CFZ was associated with 23.0% of ADRs, affecting the dermatological, ophthalmic, and gastrointestinal systems, with ichthyosis and xerophthalmia prevailing.

The use of RFM in the ROM regimen was more associated with adverse effects in the first reaction than when compared to its use in the MDT/MB regimen (*p* < 0.0001). Thus, it is possible to observe that this drug in the ROM regimen causes more adverse reactions. The average time for induction of reactions was 154 days in both therapies. This may be due to the other drugs in the MDT/MB regimen causing adverse reactions before RFM induces them. In the MDT/MB regimen, this drug caused 9.7% of total reactions, including acute renal failure, thrombocytopenia, flu-like syndrome, interstitial nephritis, headache, generalized rash, and anaphylaxis. Franco (2014) mentioned that RFM caused 30.3% (36/119) cases of flu-like syndrome and 2.5% (3/119) of DRESS, as well as 1 case of acute renal failure, similar to some cases observed in this study. No studies on adverse reactions in the ROM regimen were found, thus limiting further discussions on the ADRs of RFM use in the ROM regimen.

In Goulart et al. (2002), it was also found that the use of RFM accounted for 6.2% of the 113 reactions, affecting the renal, dermatological, gastrointestinal, and systemic systems, with fever and renal colic being the main observed reactions, as well as the renal system reactions in the current study.

Regarding the ROM regimen, there is an association with causing only one adverse reaction (*p* = 0.020), as well as gastrointestinal effects (*p* < 0.0001), mild severity (*p* < 0.0001), and possible causality according to the Naranjo Algorithm and WHO Causality (*p* < 0.001). No references were found regarding the analysis of adverse reactions to the ROM regimen.

OFX and MNC were the drugs that had the longest average days to develop an adverse reaction, at 245 and 470 days, respectively. This may be because these drugs are administered monthly, requiring more time to show ADRs. Despite OFX accounting for 7.7% (8/103) of total reactions related to the nervous, dermatological, and gastrointestinal systems, this drug can cause QT interval prolongation (Briasoulis et al., 2011), rhabdomyolysis, and tendinopathies (Hsiao et al., 2005; Alves et al., 2019), which were not identified. While MNC is known to cause lupus-like reactions (Knowles et al., 1996; Schlienger et al., 2000) and cutaneous hyperpigmentation (Hu et al., 2012), in this study, it caused only 1.9% (2/103) of reactions, associated with urticaria and itching on the scalp. Nair (2018) reported not observing adverse reactions caused by OFX and MNC in patients treated for leprosy in an alternative scheme consisting of CFZ+OFX+MNC.

The occurrence of adverse reactions is a medication-related problem, facilitating a decrease in medication adherence. Furthermore, even if a patient experiences a reaction and it is managed without discontinuing the medication, there is a greater likelihood of treatment adherence. In the context of leprosy treatment, this is of great importance because if the patient no longer uses the medication due to an adverse reaction, it can lead to relapse and resistance, complicating treatment and interruption disease transmission.

No studies have been found so far that compare the MDT/MB and ROM regimens in terms of safety, the risk of developing adverse reactions, and the average time for these effects. However, based on the results found in this study, it can be concluded that the use of the MDT/MB regimen can cause adverse reactions earlier, and it was responsible for a large part of the severe reactions, including the only fatal outcome. Regarding the ROM regimen, even though ADRs were associated with gastrointestinal effects, they were classified as mild and managed with pharmacological measures without the need to discontinue the drugs.

Thus, before starting leprosy treatment, it is necessary for the patient to undergo a detailed anamnesis with information about previous allergies and family predispositions, as well as laboratory tests to avoid confusion in variables associated with ADRs. In addition, treatment monitoring by the health team should be carried out for early identification, management, and timely treatment of adverse reactions, as well as notifications to the health authorities responsible for pharmacovigilance in each country.

Feeding the global database benefits the health of populations by ensuring the safety of medication use, leading to a reduction in morbidity and mortality, improving the rational use of drugs, and promoting safe prescription practices by healthcare professionals.

The limitation of the study was due to data collection relying on secondary means, depending on the professionals’ records, which often lacked prior knowledge for the identification of ADRs, leading to underreporting. As a commitment of the study regarding the information on these reactions, all ADRs were reported to ANVISA, Brazilian regulatory agency, via VigiMed.

Additionally, regarding limitations, this retrospective cohort study prioritized the examination of prognostic factors over the assessment of efficacy and effectiveness, distinguishing it from quasi-experimental designs and clinical trials. As mentioned earlier, the constraints within our secondary data presented challenges in obtaining comprehensive information regarding patients who did not experience drug reactions. This lack of data is crucial for quantitatively calculating relative risk and effectiveness in a comprehensive manner.

## Data Availability

The original contributions presented in the study are included in the article/Supplementary Material, further inquiries can be directed to the corresponding author.
