# Supplementary material for: Adverse reactions induced by MDT/WHO (Rifampicin+Clofazimine+Dapsone) and ROM (Rifampicin+Ofloxacin+Minocycline) regimens used in the treatment of leprosy: a cohort study in a National Reference Center in Brazil
Source: Front Pharmacol. 2024 Mar 7;15:1346169. doi: 10.3389/fphar.2024.1346169 (PMC10955366; doi:10.3389/fphar.2024.1346169)
Supplement: Supplementary file 1 [file Table1.DOCX]

Supplementary Material

## Supplementary Table

**Supplementary Table 1.** Exams performed by study patients according to the frequency in which they were requested, or with patients' needs, including to prove and monitor adverse reactions.

| Frequency of exams | Exams |
| --- | --- |
| 3/3 months | Complete blood count, fasting plasma glucose, urea, creatinin, AST, ALT, bilirubin (total), gamma globulin, alcaline phosphatase and lactic dehydrogenase and others exams if necessary, according with each individual case. |
| Treatment start and end of treatment | Complete blood count, fasting plasma glucose, colesterol levels (HDL and LDL), urea, creatinin, AST, ALT, bilirubin (total), gamma globulin, alcaline phosphatase, lactic dehydrogenase, serum calcium, serum magnesium, serum phosphorus, serum sodium, albumin, C reactive protein, serum iron, uric acid, total iron-binding capacity, tranferrin saturation, serum ferritin, folic acid, TSH, free T4, LSH, total testosteron, total PSA (if man), vitamin B12, vitamin D, HbA1c, thrombin time, erytrhrocyte sedmentation rate, IgG and IgM anticardiolipin, anticoagulant lupus, ANA, rheumatoid fator, HBSAG, Anti-HBS, Anti-HCV, Anti-HIV I/II, Anti HBC IgG, VDRL, *T. cruzi* immunofluorescence, urinalysis, parasitological examination. |
